# Dominique Soldati-Favre: Bringing *Toxoplasma gondii* to the Molecular World

**DOI:** 10.3389/fcimb.2022.910611

**Published:** 2022-05-27

**Authors:** Joana M. Santos, Karine Frénal

**Affiliations:** ^1^Université Paris-Saclay, CEA, CNRS, Institute for Integrative Biology of the Cell (I2BC), Gif-sur-Yvette, France; ^2^Université Bordeaux, CNRS, Microbiologie Fondamentale et Pathogénicité, UMR 5234, Bordeaux, France

**Keywords:** apicomplexa parasite, *Toxoplasma*, pioneer, parasitology, breakthrough, molecular parasitology

## From RNA Processing to Parasite Biology

Anyone that knows Dominique, knows that she has a real passion for science and discovery. This passion includes parasitology so it might come as a surprise that she moved to the parasitology field almost by accident.

After doing a brilliant PhD in Zurich, Switzerland, in the Schümperli team, working on RNA processing, Dominique, with the support of a Swiss National Foundation postdoctoral grant and an EMBO fellowship, decided to join a lab in the US East Coast, but life decided otherwise and, in 1991, she joined John Boothroyd’s team at Stanford University, in California. This was an outstanding team, with access to exceptional facilities, but it was transitioning from working on Trypanosomes to studying *Toxoplasma gondii*. Although *Toxoplasma* was easily cultured in the lab, it had yet to be genetically modified. At a time when the genome had yet to be sequenced and molecular biology was done without kits, Dominique, with her high energy level and swiss organization, showed that *Toxoplasma* could be transfected, a first for an intracellular parasite (Soldati and Boothroyd, 1993). She also showed, with other team members, that the transfected parasites could be drug selected and used for transgene expression, gene knockout and complementation (Kim et al., 1993; Soldati and Boothroyd, 1995; Black et al., 1995; Soldati et al., 1995). Her work did an enormous amount for the field and things became easier afterwards.

In 1995, Dominique finished her postdoctoral studies and moved to Germany to become an independent group leader. She was appointed Assistant Professor at the Center for Molecular Biology at the University of Heidelberg. There, she adapted the Cre-loxP system to *Toxoplasma* (Brecht et al., 1999) and revolutionized the field yet again by establishing the first inducible knockdown system for an Apicomplexa (Meissner et al., 2001; Meissner et al., 2002b). In 2001, Dominique moved her team to the Imperial College London, in the UK, where she held a position as a Senior Lecturer and Reader. In 2004, she became a Visiting Professor in parasitology at Imperial College and was appointed Associate Professor at the Faculty of Medicine of the University of Geneva, where her team is still based at, and where she became a Full Professor in 2010.

## Understanding *Toxoplasma* at the Molecular and Structural Level

Dominique’s lab is one of the most celebrated teams in the Apicomplexa and parasitology field, known for combining technology with biology to answer specific questions, to be highly productive and for focusing on many different aspects of *Toxoplasma* biology with endless curiosity. Dominique never “fears to enter new territory and/or to challenge old dogma that might not be right”. The Soldati team has contributed immensely to our understanding of parasite motility, host cell invasion and egress, protein trafficking, energy metabolism and even mice behavior. Two long-term research topics of the lab have been investigation of the actomyosin machinery powering gliding; and microneme composition, secretion and biogenesis. In the last decade, the Soldati lab has also explored the metabolism of both *Toxoplasma* and *Plasmodium*, as well as other molecular aspects of the malaria parasite.

Gliding motility and invasion of Apicomplexans were known to be active processes powered by a parasite actomyosin system (Dobrowolski and Sibley, 1996; Dobrowolski et al., 1997) but Dominique’s lab was the first to identify its molecular components. In an impressive endeavor, her team characterized the kinetic and mechanical properties of the myosin heavy chain protein A (TgMyoA) with protein directly purified from tachyzoites (Herm-Götz et al., 2002), a first for a myosin. Simultaneously, they demonstrated that TgMyoA is critical for parasite motility and host cell invasion (Meissner et al., 2002b). Finally, the name “glideosome” was proposed by Dominique’s lab to describe this new and unique actomyosin system (Opitz and Soldati, 2002). Together with other labs, the team then identified and functionally characterized the glideosome components (Gaskins et al., 2004; Frénal et al., 2010; Nebl et al., 2011; Williams et al., 2015; Jacot et al., 2016), as well as regulators of actin dynamics (Plattner et al., 2008; Mehta and Sibley, 2010; Daher et al., 2010; Yadav et al., 2011; Salamun et al., 2014; Jacot et al., 2016). Later, identification of Myosin H (TgMyoH), actin nucleator Formin 1 (TgFRM1) and the glideosome-associated connector (TgGAC) at the tachyzoites conoid showed how gliding motility is initiated at the parasite’s apical tip (Graindorge et al., 2016; Jacot et al., 2016). Finally, very recently, the lab used expansion microscopy to explore the apical complex structure and function, including that of the enigmatic conoid (Dos Santos Pacheco et al., 2021).

In parallel, Dominique’s lab has had a continued interest on micronemes, specialized parasite apical secretory organelles, storing adhesins and other effector molecules implicated in gliding motility, host cell attachment, invasion and egress. The lab has identified several of its components and protein complexes, and investigated their trafficking, structure and function during the parasite lytic cycle (Di Cristina et al., 2000; Ferguson et al., 2000; Reiss et al., 2001; Brecht et al., 2001; Meissner et al., 2002a; Huynh et al., 2003; Mital et al., 2005; Blumenschein et al., 2007; Kessler et al., 2008; Friedrich et al., 2010; Sheiner et al., 2010; Marchant et al., 2012; Sardinha-Silva et al., 2019). In recent years, the team ventured into discovering the mechanisms behind microneme biogenesis and exocytosis. In 2018, they identified Transporter Facilitator Protein 1 (TgTFP1) (Hammoudi et al., 2018), an essential protein for parasite survival due to its crucial role in microneme biogenesis and maturation. In 2016, the identification of the protein acylated pleckstrin homology domain-containing protein (TgAPH) as a phosphatidic acid sensor anchored at the surface of the micronemes (Bullen et al., 2016) prompted the lab to investigate the signaling cascade leading to microneme secretion and parasite egress (Bullen et al., 2016; Jia et al., 2017; Darvill et al., 2018; Bisio et al., 2019; Bisio et al., 2022) and, with other labs, to draw the picture we know today (Farrell et al., 2012; Brown and Sibley, 2018; Bisio and Soldati-Favre, 2019; Bullen et al., 2019; Yang et al., 2019).

Another topic frequently addressed by Dominique throughout the years has been parasite metabolism. She was one of the first in the field to propose that energy metabolism could be exploited therapeutically (Polonais and Soldati-Favre, 2010). Ever since, her team has defined the parasite metabolic needs and capabilities by using both *in silico* and *in vivo* approaches to investigate specific metabolic pathways (Limenitakis et al., 2013; Oppenheim et al., 2014; Kloehn et al., 2020; Kloehn et al., 2021) but also build metabolic models for *T. gondii* tachyzoite and bradyzoite stages (Tymoshenko et al., 2015; Krishnan et al., 2020).

The Soldati team has also ventured into studying *Plasmodium*, in collaboration with other teams but also solo, with as much success as with *Toxoplasma* (Pino et al., 2012; Chiappino-Pepe et al., 2017; Stanway et al., 2019; Bisio et al., 2020; Bertiaux et al., 2021). They have, for instance, identified a new multistage antimalarial inhibitor blocking both parasite invasion and egress (Pino et al., 2017) and investigated specific metabolic pathways of *P. berghei* intra-erythrocytic and liver stages (Oppenheim et al., 2014; Stanway et al., 2019).

## Recognized by Her Peers

Parasitology is a small and relatively neglected field compared to others so even those who are exceptional are rarely praised. Even so, Dominique has been the recipient of multiple accolades, namely the Kar Asmund Rudolphi-Medal of the German Society for Parasitology in 2001, the Prize of the Gertrude von Meissner Foundation in 2009 and twice the Pfizer Prize for Basic Research in Infection, awarding specific lab publications, and the Cloëtta Foundation Prize in 2015 and the Alice and C. C. Wang Award in Molecular Parasitology in 2019, acknowledging Dominique’s scientific career. She is a member of prestigious academies, including the Swiss Academy of Medical Sciences, the European Academy of Microbiology and EMBO, was an HHMI International Scholar and Senior Scholar in Infectious Diseases, and she has received a number of prominent grants, including a European Research Council (ERC) Advanced Grant, unarguably one of the most competitive European grants. She has also organized numerous parasitology meetings, including the Molecular Parasitology Meeting or the Gordon Research Conference on Host-Parasite Interactions, she is the Academic Editor of selected publications for various journals (eLife, PLoS Pathogens, Traffic, Cell Host & Microbe, and others), she has been a contributor to the F1000 since 2006, and she has been a grant reviewer for national and international grant schemes and an expert for the ERC or the Wellcome Trust’s Peer Review College.

Despite all the praises Dominique has received, and all the high impact papers she has published, many of the people we interviewed mentioned that they didn’t actually know who Dominique was when they interviewed - it was “after talking to her in her little office that it became immediately clear to me that I absolutely wanted to join her lab - her personality and how she talked about science and her research inspired me from the minute I met her”, it was “only after spending a few months in her lab that I realized how lucky I was!”. This is a testament of Dominique’s ability to never wanting to be the bigger person in the room and for always caring for the person in front of her - “she took a huge interest in my future career and supported me wherever she could”, “I had always the freedom to make my own decisions and she would respect them”.

“Dominique is firm when people want women to be nice”, someone said. Indeed, Dominique is straightforward and says exactly what she thinks. Despite what some called a steep learning curve, everyone was unanimous in saying that Dominique masters the art of getting everyone to push themselves and be motivated. From the very beginning of her career as a mentor, Dominique was certainly demanding but never pressured people to work harder or gave the impression that she thought that way (even if she might…).

Dominique always let people do side projects, think for themselves. Her enthusiasm and energy are an unlimited source of motivation that pushed us all to work more and be better. Some of us even remember presenting to her results of experiments done behind her back and having Dominique being very encouraging and open for new, even crazy ideas. This is certainly a result of the lab good atmosphere, which has lasted, even though the team has moved internationally twice. As a postdoc, Dominique was known to have fun and even played some unforgettable jokes on the lab head. We can say this trait has continued. The lab beer o’clocks and the department dress up Christmas parties were always memorable.

## Representation and Empowerment

This essay is written in the context of a special issue of Women in Parasitology. Dominique would be the first to say that she is not a woman scientist but a scientist that is also a woman. When she became a group leader, there were not many women in that position and Dominique herself has said that the low number of women in the field was one of the obstacles she found in her career. While the number of women in the field has increased, men still dominate, especially at high positions. Women we interviewed said that working with Dominique was empowering as a woman not because of something she said or did in particular but because just seeing another woman doing well in your field is empowering.

Throughout her career, Dominique underwent changes family-wise, going from being a mother of 2 during her postdoc, to having 3 and then 4 children as a lab head. To her, family was at the center of her choices without being a barrier and that showed us that it doesn’t have to be one or another but we can do both and do it well. It was always motivating to observe her living for science even if we all knew she had a family life outside the lab.

Dominique has been an example not only to the people that worked/work with her but to anyone that has interacted with her at workshops, training courses or conferences. At the University of Geneva, she has been vice-dean for research at the Faculty of Medicine for 10 years, she is the head of the department of Microbiology and Molecular Medicine since 2020 and, since 2008, she is the director of the Graduate School Biomedical Sciences. She has also taught numerous workshops, including a Cell biology workshop in Mali in 2012 and the Biology of Parasitism (BoP) and Middle-Eastern BOP courses. During these courses, Dominique always took the time to discuss with every student and was keen to learn about their culture, career, and ambitions. Dominique is also a mentor for women outside science, as part of the Swiss-French network for mentoring women careers.

## Final Thoughts

**Figure 1:**
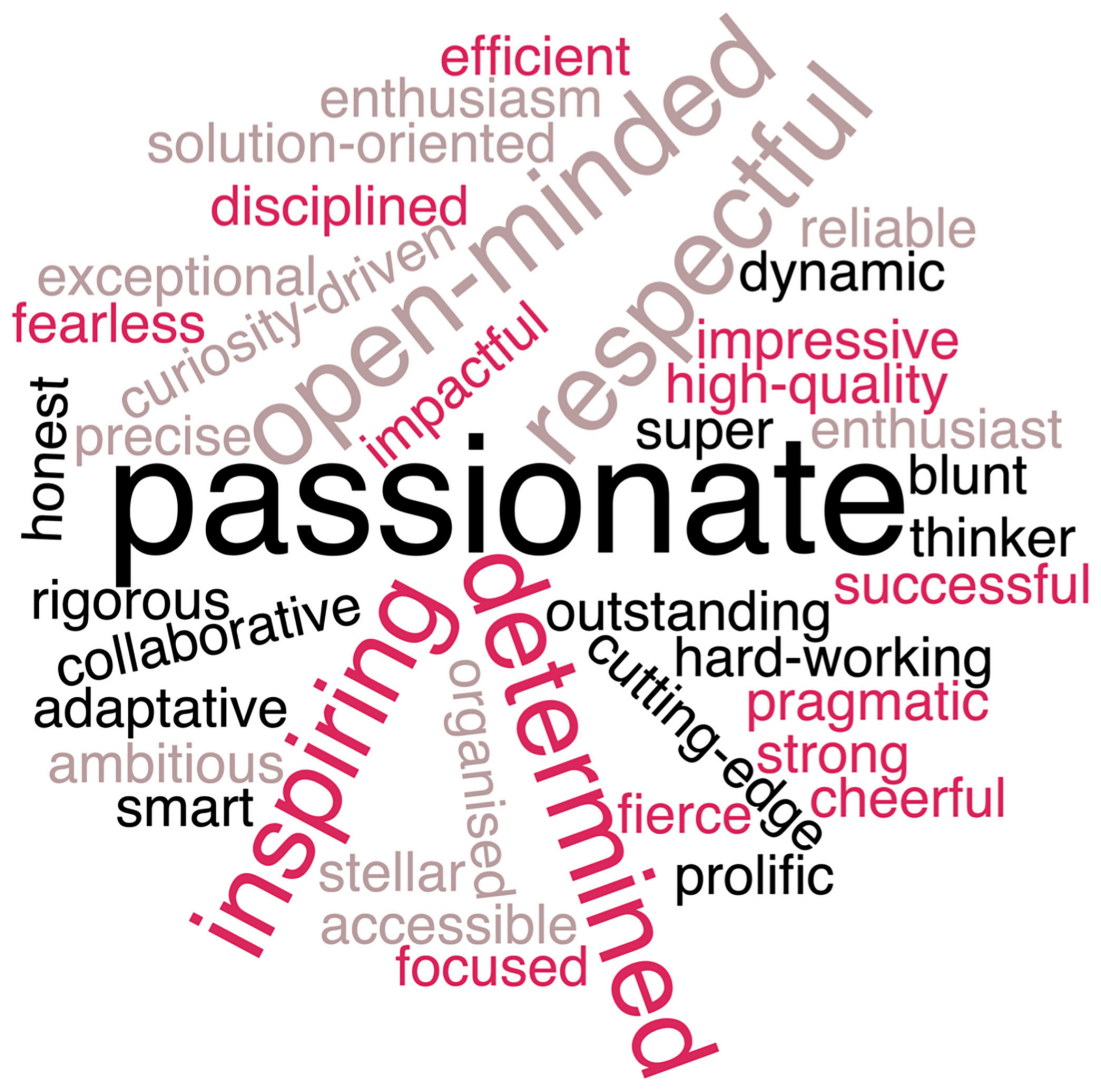
Words used by former supervisor, colleagues, PhD students and post-docs to describe Dominique’s personality, career and research.

The words used by her supervisor, colleagues, former students and postdocs to describe Dominique’s career, personality and research are shown on **Figure 1** but we think that more than anything we can write, some of what was shared with us better exemplify the impact she has had on all of us: “I looked for a good supervisor and mentor. Dominique was both from the very beginning”, “I am proud to have been in her lab”, “Dominique represents the person combining all the capacities to direct and lead research projects and above all the capacity to transmit these values”, “Dominique has undoubtedly shaped my way of doing science”, “Dominique has been the only PI who I felt really cared about her people in the lab and would ensure everyone was on the right track”, “Dominique’s work pushes us to do better science”, “Dominique is an inspirational colleague, rigorous in her science”.

We hope this essay highlights the pioneer and breakthrough work Dominique has done and continues to do but also what a role model she has been for all of us who have had the privilege to work with her. Both men and women see Dominique as a mentor and as an inspiration in the way she does science, shares her knowledge, runs the lab and is available even though she also has a family, travels frequently and has to attend to other professional obligations.

## Author Contributions

Both authors prepared, wrote and edited the manuscript and jointly made the figure.

## Conflict of Interest

The authors declare that the research was conducted in the absence of any commercial or financial relationships that could be construed as a potential conflict of interest.

## Publisher’s Note

All claims expressed in this article are solely those of the authors and do not necessarily represent those of their affiliated organizations, or those of the publisher, the editors and the reviewers. Any product that may be evaluated in this article, or claim that may be made by its manufacturer, is not guaranteed or endorsed by the publisher.
